# Role of Time Scales in the Coupled Epidemic-Opinion Dynamics on Multiplex Networks

**DOI:** 10.3390/e24010105

**Published:** 2022-01-09

**Authors:** Robert Jankowski, Anna Chmiel

**Affiliations:** 1 Faculty of Physics, Warsaw University of Technology, Koszykowa 75, 00-662 Warsaw, Poland; anna.chmiel@pw.edu.pl; 2 Departament de Física de la Matèria Condensada, Universitat de Barcelona, Martí i Franquès 1, 08028 Barcelona, Spain; 3 Universitat de Barcelona Institute of Complex Systems (UBICS), Universitat de Barcelona, 08028 Barcelona, Spain

**Keywords:** multiplex networks, epidemic spreading, COVID-19, opinion dynamics, voter model

## Abstract

Modelling the epidemic’s spread on multiplex networks, considering complex human behaviours, has recently gained the attention of many scientists. In this work, we study the interplay between epidemic spreading and opinion dynamics on multiplex networks. An agent in the epidemic layer could remain in one of five distinct states, resulting in the SIRQD model. The agent’s attitude towards respecting the restrictions of the pandemic plays a crucial role in its prevalence. In our model, the agent’s point of view could be altered by either conformism mechanism, social pressure, or independent actions. As the underlying opinion model, we leverage the *q*-voter model. The entire system constitutes a coupled opinion–dynamic model where two distinct processes occur. The question arises of how to properly align these dynamics, i.e., whether they should possess equal or disparate timescales. This paper highlights the impact of different timescales of opinion dynamics on epidemic spreading, focusing on the time and the infection’s peak.

## 1. Introduction

The work by Kermack and McKendrick [1] is acclaimed as the primary mathematical modelling tool of infectious diseases. The so-called SIR model considers a fixed population with only three compartments: S (Susceptible), I (Infected), R (Removed), with a fixed flow of states, and is described as a system of differential equations. Despite its simplicity, the model can be used to highlight the importance of social distancing and safety measures such as using face masks or hand-washing. By incorporating these health-prevention recommendations, we could mitigate the disease spread, i.e., reduce the infection probability [2]. That strategy is known as “flattening the curve“ and prevents a health care system from being overwhelmed.

On the other hand, the SIR model, in its general form, lacks many real data features, e.g., assumes homogeneous contact probability and unidirectionality. Recent advances in network science have shown key applications in epidemic-spreading processes [3]. With the underlying graph structure of contacts, we introduce the heterogeneity of infection probability based on the degree of a given node. Epidemic spreading has been studied on scale-free networks [4], hierarchical social networks [5], networks with community structure [6], and correlated [7] or weighted [8] complex networks. All of these works operate on the single-layer network.

Ubiquitous access to information prevents us from examining the epidemic spread without considering other important aspects, e.g., social or economic aspects. A similar reasoning led to the introduction of the multilayer concept into network science—over the last 15 years, the use of multiplex networks has become increasingly popular in modelling complex human behaviour [9,10,11,12]. Notable work on the connection between epidemic spreading and awareness has been carried out by Granell et al. [13]. They studied the interrelation between two processes responsible for epidemic spreading and the information awareness to prevent its infection, on top of multiplex networks. It is interesting to note that the critical point for epidemic outbreaks is defined by the awareness dynamics and the topology of the virtual network.

The interplay between awareness and epidemic spreading could be studied in the very early phase of the epidemic. At present, due to the omnipresence of social media, shortly after the epidemic outbreak, society was already informed and aware of the current situation. This is in contrast, for example, to the Spanish flu, a deadly global influenza pandemic dated 1918 [14], when people from different cities were largely unaware of the fact that the epidemic was in progress. Here, we focus on another aspect, i.e., the opinion of restrictions caused by the epidemic. According to the World Health Organization (WHO), to protect ourselves and prevent the spread of COVID-19, one needs to (i) wear a correctly fitted mask if physical separation is not possible and in poorly ventilated environments, (ii) wash hands frequently with alcohol-based hand rub or soap and water, (iii) cover one’s mouth and nose with a bent elbow or tissue when one coughs or sneezes, (iv) self-isolate until recovery in the case of symptoms or testing positive for COVID-19 [15]. These regulations are not limited to the COVID-19 pandemic; they were also obeyed in the H1N1 influenza [16] or Ebola virus outbreak [17]. Wearing face masks is one of these safety precautions and has been corroborated to mitigate disease prevalence [18,19]. However, some individuals express an opposite opinion, forming a so-called anti-mask minority [20]. These attitudes could lead to a strongly polarized society [21] and affect the epidemic’s pace [22]. Hence, this work is dedicated to scrutinizing the interrelationship between opinion dynamics and epidemic spread. We would like to emphasise that the issue of the COVID-19 infodemic [23], leveraging the threats of spreading misinformation (especially on social media [24]), is acknowledged as one of the possible factors that could devastate the effectiveness of public health measures.

In this study, we adopt the so-called *q*-voter model [25] as the underlying opinion dynamic. It is a binary opinion model with special cases of both the linear voter model [26] and the Sznajd model [27]. Nyczka et al. [28] investigated *q*-voter model in the presence of different types of nonconformity and showed the differences between the two types of stochastic noise, anticonformity and independence, that play a crucial role in the phase transition observed in the system. In the case of anticonformity, the critical value of noise increases with the parameter *q*, whereas in the model with independence, the critical value of noise decreases with *q*. The character of the phase transition strongly depends on the type of noise in the model: with anticonformity, the phase transition is continuous for any value of *q*, whereas in the model with independence, the transition is continuous for q≤5 and discontinuous for q>5. A comprehensive mathematical description of the *q*-voter model behaviour in the complex networks was obtained by applying the pair approximation approach [29]. Although it is a straightforward model, it has been applied to the characterization of various dynamical phenomena, such as the diffusion of innovation [30] or recurring fashion cycles [31]. It is also worth mentioning that, when considered in duplex and multiplex settings, the *q*-voter model provides a very rich behaviour with respect to the observed phase transitions [32,33]. Considering these observations, we eventually select the *q*-voter model due to its apparent simplicity and low number of tunable parameters that can lead to complex results.

With two distinct dynamics, epidemic spreading and opinion prevalence, the question arises of whether to treat them equally, i.e., run one opinion update per one epidemic update. Usually, one assumes that the two dynamics possess the same timescales [34,35,36,37]. However, the transmission of opinion could be significantly faster than the spread of disease due to common access to the Internet. Recent works highlight the impact of timescales in the spread of interacting diseases [38] or awareness dissemination [39]. For instance, in [38] the authors inspected asymmetrically interacting diseases and concluded that if a dominant disease has a faster clock, the prevalence of both diseases decays, possibly eradicating the weaker one. On the other hand, in [39], the authors considered the interplay between information propagation and epidemic spreading, outlining that the timescales between the information and infection processes determine whether information awareness is beneficial for the magnitude of the epidemics. These facts motivate us, in this study, to deepen the knowledge of the role of timescales in the context of coupled opinion–epidemic dynamics. The main aim of the work is to highlight the importance of the time dependence (separation of scales) between two classes of dynamical processes, i.e., the social and the epidemic classes. We add a simple mechanism of the relative rate of dynamics, which results in the level of opinion being “faster” than the epidemic class. We expect that, when the relative rate grows, the influence of the epidemic dynamic will become less pronounced for the agents’ initial opinions.

## 2. Materials and Methods

**Figure 1:**
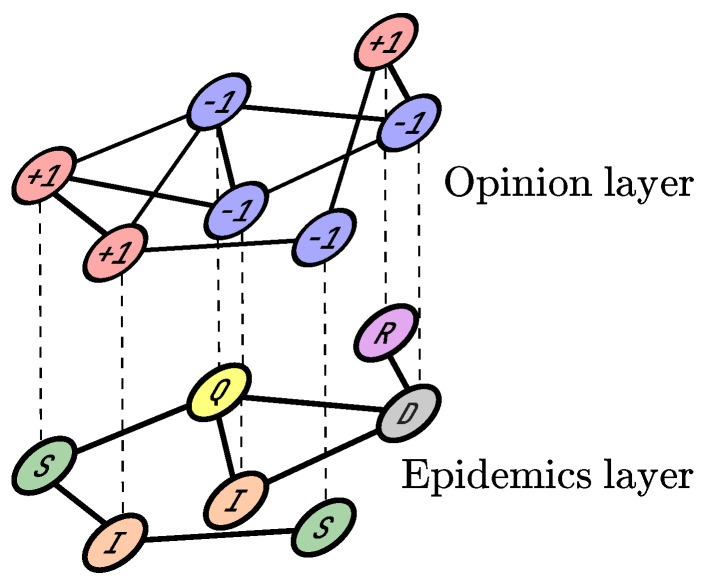
Representation of the opinion–epidemic model. The upper (opinion) layer considers opinion dynamics, and nodes possess two possible states: positive (+1) or negative (−1). This layer also contains additional connections between agents. The lower (epidemic) layer supports the spread of disease. The nodes are the same agents as in the opinion layer, but their states can be (S) susceptible, (I) infected, (Q) quarantined, (D) deceased or (R) recovered.

Our model considers the epidemic spread of the disease alongside the propagation of opinions respecting restrictions and regulations. Individuals could impede the prevalence of the epidemic by maintaining social distancing, wearing face masks, or handwashing. Two dynamics operate on a double-layer multiplex network that forms the opinion–epidemic model (see Figure 1).

In the first layer, we leveraged the *q*-voter model, which many scholars have studied extensively [33,40,41,42,43]. In our context, this assumes that each agent *i* has an opinion respecting the current restrictions, given by a binary variable: $S_{i}\left(t\right)=+1\left(o_{+}\right)$ or $S_{i}\left(t\right)=-1\left(o_{-}\right)$. This describes either a positive or negative view towards compliance with the rules. We only allowed individuals who have a fully supportive opinion, i.e., agents either respect all current restrictions (social distancing, wearing face masks and handwashing), or truly disagree and do not follow any of the rules. At each elementary update, we randomly selected a node *i* from the entire system. An agent in a given update could behave in one of two ways. It acts independently with a probability *p*, or it acts like a conformist, with complementary probability 1−p. In the first case (independence), an agent is unwilling to yield to group pressure, and flips to the opposite opinion. In the second case (conformism), an agent *i* is influenced by a group of size *q* (randomly chosen), and that agent adapts to the group only if the opinion is unanimous. Otherwise, the opinion of the agent *i* remains the same.

In the second layer, we consider the SIQRD model [44], where each node can be in one of five distinct states: (S) susceptible, (I) infected, (Q) quarantined, (R) recovered or (D) deceased, at a given timestep. In contrast to the original SIR model, the Removed state is split by differentiating among the Recovered and Deceased individuals; additionally, we account for the intervention procedure to control the spread of the disease in the form of quarantine (Q, see, e.g., [45])—this compartment played a crucial role in the recent COVID-19 pandemic [46]. Although, in this study, we shall focus mainly on the evolution of the number of infected individuals, perhaps making the split between R and D seem superficial, we underline that agents in the D state are unable to further evaluate their opinion (which is not true in the case of Recovered agents).

We want to emphasize that this work does not aim to develop a prediction model for COVID-19 but to explore a salient, yet usually neglected, aspect of the interplay between opinion dynamics and epidemic prevalence. In recent years, advances in technology have allowed us to gather precise information about infection statistics. Thus, the data from the onset of COVID-19 to the current day are easily accessible, and some could serve as an appropriate estimate of the initial parameters of our model. In particular, the European Centre for Disease Prevention and Control reports an infection time duration $t_{i}$ between 5 and 14 days for COVID-19 [47]. Therefore, in addition, each agent remains in the infected state for $t_{i}$ timesteps, where $t_{i}$ is sampled from normal distribution with μ=10 and σ=5. Moreover, the distribution is restricted to non-negative values.

**Figure 2:**
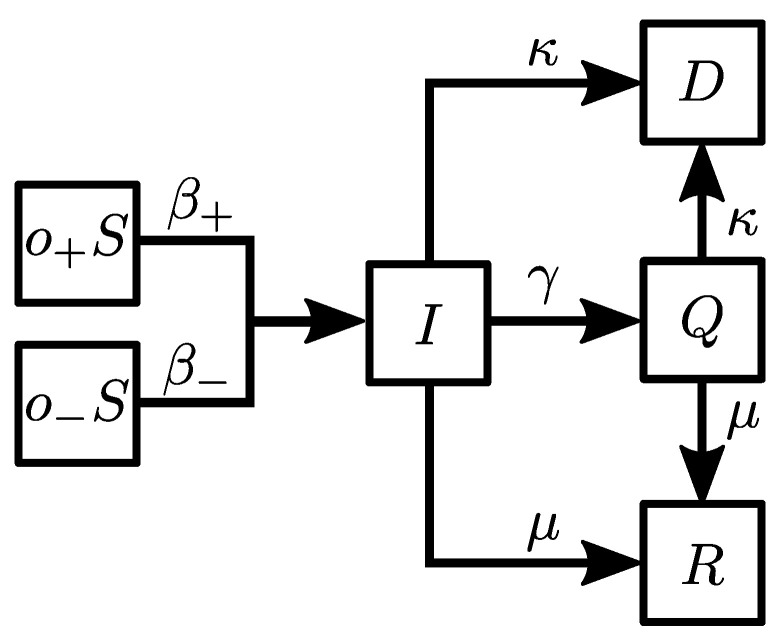
Schematic representation of agent states with associated transition probabilities.

We allow the following transitions between epidemic compartments (see Figure 2).

(i) $S\overset{\beta }{\to }I$: a susceptible agent becomes infected with the probability β.
(ii) $I\overset{\gamma }{\to }Q$: an infected agent goes into quarantine with the probability γ.
(iii) $I\overset{\mu }{\to }R$: an infected agent recovers with the probability μ.
(iv) $I\overset{\kappa }{\to }D$: an infected agent dies with the probability κ.
(v) $Q\overset{\mu }{\to }R$: an agent in quarantine recovers with the probability μ.
(vi) $Q\overset{\kappa }{\to }D$: an agent in quarantine dies with the probability κ.

Here, we introduce the interplay between these two layers. First, a susceptible agent with a positive opinion ($o_{+}S$, see Figure 2) is more prone to respect the restrictions. Thus, the probability of being infected in this case is decreased by half, i.e., $\beta_{+}=\beta /2$. On the other hand, the infection probability for the agent with a negative opinion ($o_{-}S$) remains the same $\beta_{-}=\beta$. Second, we reduce the duration of the infected state for the positive agent $t_{i}\left(o_{+}\right)=t_{i}/2$ and keep the same duration for the agent with a negative opinion. People who comply with the rules are willing to limit their level of social contact and stay in quarantine [22]. One could consider separating the transition probability to the quarantine state for agents with positive and negative opinions, indicating that individuals with positive attitudes are more eager to isolate themselves. However, at present, it is not uncommon for governing bodies such as the Ministry of Health to impose global restrictions regarding quarantine. Hence, all individuals possess the same rate of entering quarantine.

Here, we use Holme et al.’s network [48] as the underlying topology of agent–agent interactions. It is a modified version of the Barabási–Albert (BA) network [49] with a “triad formation step”. This step produces networks with high clustering coefficients, often observed in many real systems [50]. We start from *m* disconnected nodes. In every timestep, a new node *v* with *m* edges is added. Each edge of *v* is then linked to an existing vertex, and the probability is proportional to its degree, i.e., we apply the preferential attachment (PA) rule. This probability of a node *w* being attached to *v* is given by $P_{w}=k_{w}/\sum_{v\in V}k_{v}$. In the original setting of the BA model, the growth step is repeated *N* times, and, for each growth step, the PA step is iterated *m* times for *m* edges of the newly added node. However, here, we perform an additional step, namely, if an edge between nodes *v* and *w* was created in the last PA step, then one more edge from *v* is added to a randomly chosen neighbour of *w*. If there is no pair to connect, i.e., if all neighbours of *w* were already connected to *v*, we perform a PA step instead. In the first (opinion) layer, we also add $E_{add}$ additional links, as in [13,51]. Using a scale-free network as the topology of the opinion layer introduces an issue with the size of *q*-lobby. Even though we restrict the average degree $\langle k\rangle$ in the network to be larger than the size of the group, sometimes the node does not have enough neighbours to choose from. We account for such a situation, leaving the opinion of such a node in its original state.

To study the role of timescales in a more precise manner, we introduce a parameter $v_{step}$, which controls the speed of state change in the *q*-voter model, i.e., per each timestep on the epidemic layer, we perform $v_{step}$ updates on the opinion layer. In other words, $v_{step}$ can be regarded as the relative rate between two processes.

Table 1 summarises the model’s parameters, and indicates which can be treated as a variable during experiments.

## 3. Results

To examine the above-described system, we carried out Monte Carlo simulations on multiplex networks. Each timestep in the simulations comprised *N* micro-steps, where *N* is the size of the system. We selected the number of time steps to allow the system to reach a steady state. The runs were averaged over multiple realizations to maintain a low level of error. The set of initial parameters is shown in Table 1. Due to the phase transition image in *q*-voter model (see, for instance, Figure 5 in [29]), we focused on smaller values of independence probability *p*. When *p* exceeds a certain threshold, which depends on a few factors, such as network topology and its mean degree, the mean opinion in the system converges to zero, i.e., half of the agents have a positive opinion, half have the negative one. A greater independence probability such as p=0.9 would indicate that the underlying dynamic is random to a greater or lesser degree. Sample realizations of the model before and after simulations are included in Supplementary Materials, Figures S1–S4.

### 3.1. Role of the Opinion Layer

**Figure 3:**
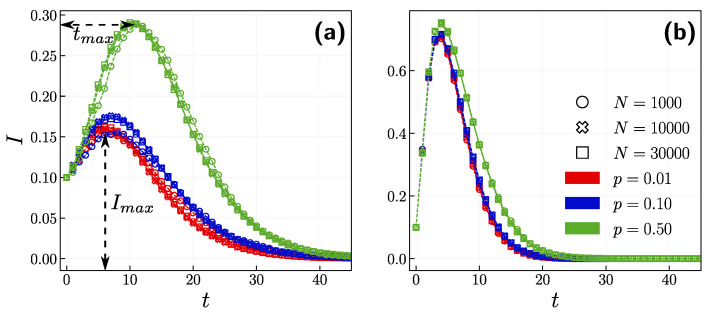
The time evolution of infection rate for different independence probability p={0.01,0.1,0.5} with N={1000,10,000,30,000}. (**a**) β=0.02, (**b**) β=0.2. We outline the peak of infection $I_{max}$ and time when it occurs $t_{max}$ in panel (**a**).

We began by scrutinizing the impact of the opinion layer on the epidemic spread. Figure 3 shows the time evolution of the infection rate I(t) for three different independence probabilities *p* and different network sizes. First, we concluded that the results do not depend on the size of the network; therefore, for the rest of the experiments, we set N=10,000. Second, when β=0.02 (Figure 3a) we observed that the peak of infection $I_{max}$ for p=0.5 is higher and occurs later than for smaller *p* values. In a situation when agents act on their own rather than following the group, $I_{max}$ increases by almost half in comparison to conformist agents. Regarding β=0.2 (Figure 3b), the peaks of infection for all values of *p* appear almost simultaneously, and their values are close to each other.

**Figure 4:**
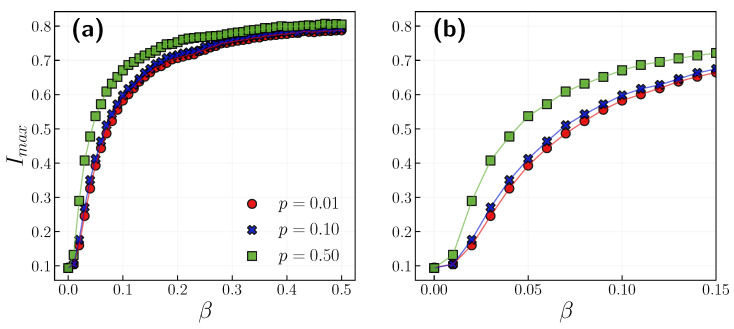
(**a**) The peak of infection $I_{max}$ in the function of infection probability β with p={0.01,0.1,0.5}. The results are averaged over 10 realizations. Error bars are smaller than the symbols’ sizes. (**b**) Close-ups of smaller β values.

Further study on the range of infection probabilities confirmed the preliminary observations. In Figure 4 one can see the infection peak in the function of infection probability for three different independence probability values *p*. The maximum infection rate grows rapidly for smaller infection probabilities up to around β=0.2, when it slowly starts to saturate. It is interesting to note that we can reduce the infection peak by 0.15 by imposing group influence, i.e., lowering *p* (see Figure 4b).

**Figure 5:**
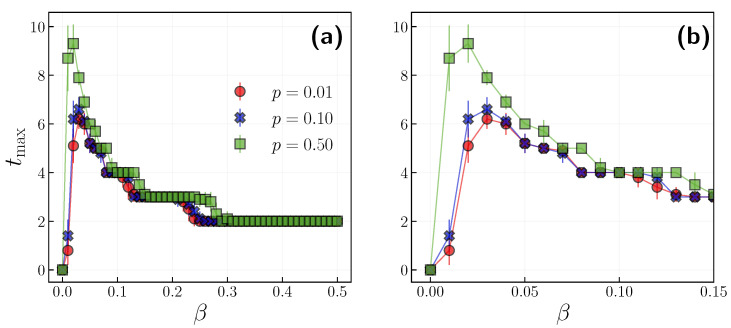
(**a**) The time of infection peak $t_{max}$ in the function of infection probability β with p={0.01,0.1,0.5}. Results are averaged over 10 realizations. (**b**) Close-up to smaller β values.

In Figure 3, we have observed that the time of the infection peak for a larger independence probability p=0.5 occurs later than the peak for smaller ones. To fully understand this relation, we measure the time of the infection peak $t_{max}$ in the function of infection probability β for three different independence probabilities. Figure 5 depicts that, indeed, for a larger independence probability, the $t_{max}$ is larger, i.e., the pace of the epidemic is slower. However, at the same time, the infection peak is larger. When the infection probability is large enough, the difference between independence probabilities becomes blurred. We note that we initially set all agents as having a positive opinion at the beginning of the simulations. A higher independence probability could be viewed as the noise in the model and induces more negative-attitude agents in the system. On the other hand, when the whole society does not respect the rules, a larger independence probability could persuade more people to hold a positive opinion, resulting in reductions in the infection peak and shortening the time for which it occurs.

**Figure 6:**
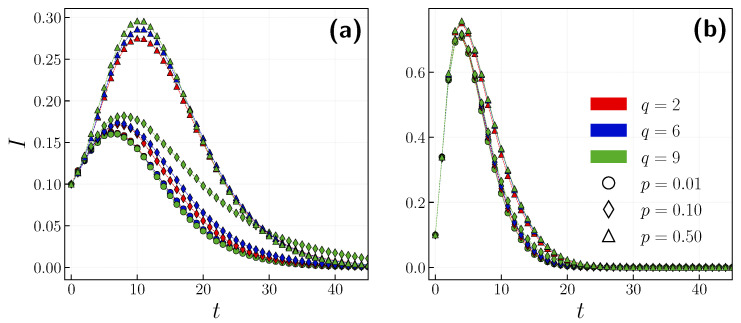
The time evolution of infection rate for different *q*-lobby size q={2,6,9} and independence probabilities p={0.01,0.1,0.5}. (**a**) β=0.02, (**b**) β=0.2.

Another important parameter of the *q*-voter model is *q*, i.e., the size of the influence group. We carried out simulations for three values of *q*, while also considering distinct independence probabilities. Figure 6 depicts the time evolution of the epidemic with the above-mentioned parameters. On the left panel, we considered β=0.02. Indeed, a larger group size has a slight impact on the epidemic trajectory. With a larger independence probability (p=0.1), a greater *q* slows down the decay of the epidemic. However, when p=0.5, the group size only impacts the peak of infection. On the other hand, when β=0.2 (see Figure 6b), the group size no longer extends the decay period or affects the peak of infection. One could argue that a stronger bond between the two layers is needed, with more infectious diseases. As we have shown in Figure 4 after exceeding a certain threshold of β, the peak of infection starts to blend for different independence probabilities. These phenomena are also present for different *q* values.

**Figure 7:**
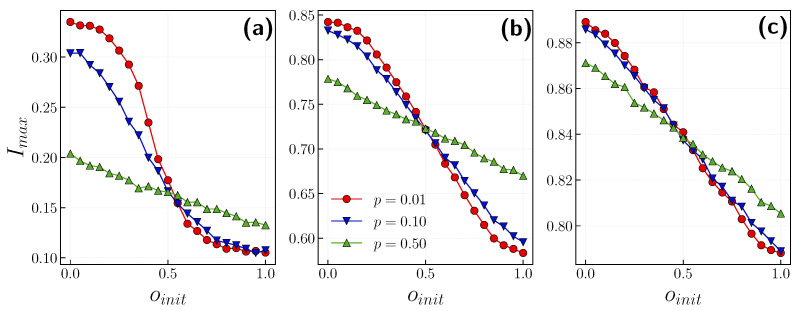
The peak of infection $I_{max}$ in the function of initial positive opinion fraction $o_{init}$ with p={0.01,0.1,0.5} for (**a**) β=0.01, (**b**) β=0.1, (**c**) β=0.5. Results are averaged over 10 realizations. Error bars are smaller than symbols’ sizes.

It was shown that the epidemic slows down in countries where people are more willing to respect the current restrictions [52]. Our model also corroborates that the peak of infection decreases when the population has a positive opinion (see Figure 7). Moreover, with a less contagious disease (smaller β), this result is more pronounced. We also show that mimicking the group could only diminish the peak of infection if that group consists of agents with positive attitudes. Otherwise, it is more suitable to stick to your opinion. However, for most contagious illnesses (β=0.5), this effect is negligible. One should note the difference between a society of agents with initially positive opinions ($o_{init}=1$) and negative ones ($o_{init}=0$) (see Figure 7a). The difference in infection peak when agents act independently (higher *p*) and remain conformist (lower *p*) is more significant for a society with only negative opinions at the beginning of the simulation. Agents who eventually change their opinion to a positive one decrease the infection probability by half and reduce the time spent in an infected state by the same amount. This effect is less pronounced in more infectious diseases since the interplay between the opinion and epidemic is weaker.

### 3.2. Role of Time Scales

Once we have understood the impact of the opinion layer on the epidemic prevalence, we can turn our attention to the role of timescales. To date, the dynamics of the two layers have been conducted in the same way. Henceforth, we consider the different speeds of opinion updates and present their effects.

**Figure 8:**
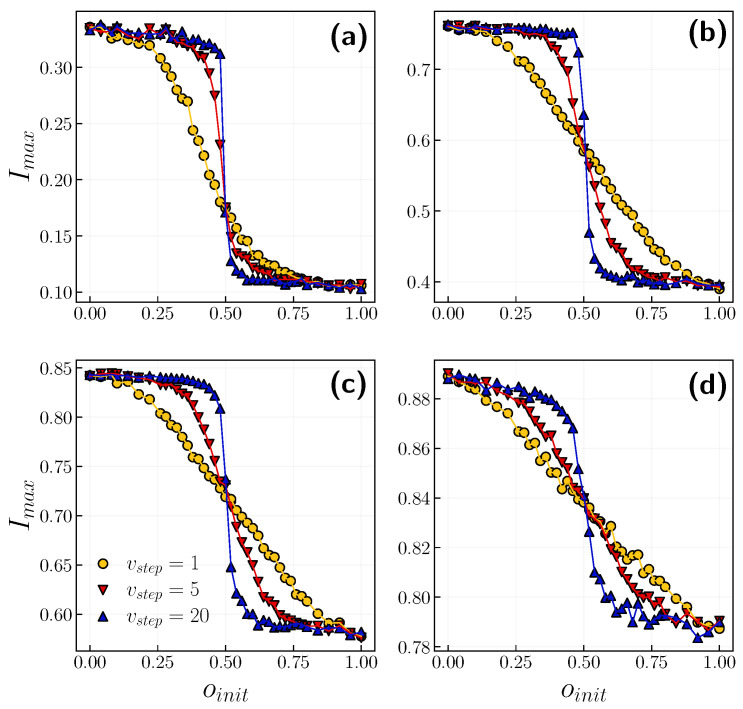
The peak of infection $I_{max}$ in the function of initial positive opinion fraction $o_{init}$ for selected timescales $v_{step}=\left\{1,5,20\right\}$ with p=0.01. Each panel corresponds to a different infection probability, (**a**) β=0.01, (**b**) β=0.05, (**c**) β=0.1, (**d**) β=0.5. Results are averaged over 10 realizations. Error bars are smaller than symbol size.

Figure 8 depicts the relationship between the peak of infection $I_{max}$ and the initial fraction of positive agents $o_{init}$ with p=0.01. For comparison, the yellow dots are the same as in Figure 7. One can observe that this relationship becomes a step function with an increasing number of opinion updates $v_{step}$. However, for a higher probability of infection, one would need to increase $v_{step}$ for the exact step function. It is worth mentioning that the initial opinion has a striking impact on less contagious illnesses. When β=0.01 (β=0.05), we can flatten the infection peak by almost 0.2 (0.4). With β=0.01, the lowest infection peak equals 0.1, since it is the initial fraction of infected agents. Hereafter, we assume that the society is unanimous and initially holds a positive opinion, i.e., $o_{init}=1$. However, we will discuss the opposite situation later.

**Figure 9:**
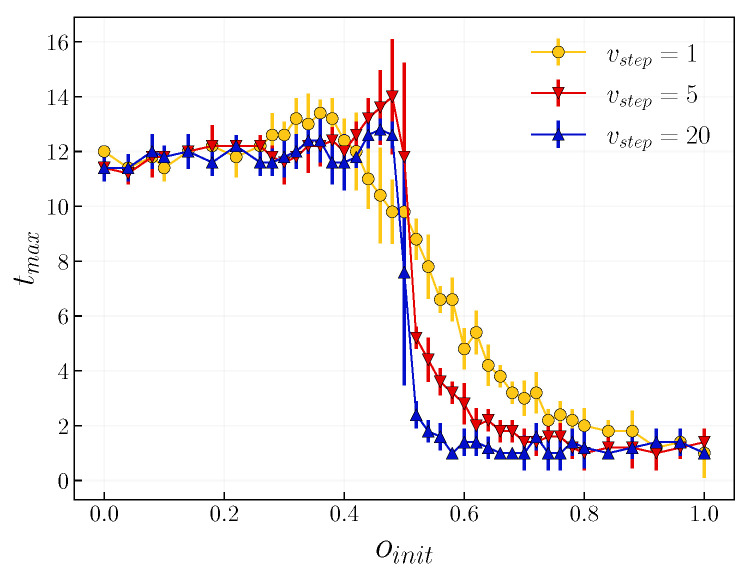
The time of infection peak $t_{max}$ in function of initial positive opinion fraction $o_{init}$ for selected timescales $v_{step}=\left\{1,5,20\right\}$ with p=0.01 and β=0.01. Results are averaged over 10 realizations.

A complementary point to consider is the impact of timescales on the time of the infection peak (see Figure 9). With equally fast dynamics, i.e., when $v_{step}=1$, the time of infection peak remains constant for a lower initial fraction of positive agents, $o_{init}$ up to 0.4, when it steadily decreases. That transition from a slower to a faster epidemic is more pronounced when we increase $v_{step}$, i.e., when the dynamic of opinion is faster. One can observe that the peak in infection for the lower initial fraction of positive agents $o_{init}$ is significantly larger than that for higher ones (see Figure 8). At the same time, the epidemic needs more time to fully develop, i.e., we have a greater $t_{max}$. When we consider a completely positive society, $o_{init}=1$, the peak of infection ranges at around 0.1, which is the initial fraction of infected. This can be seen in the results shown in Figure 9, i.e., the time of these peaks occurs immediately.

**Figure 10:**
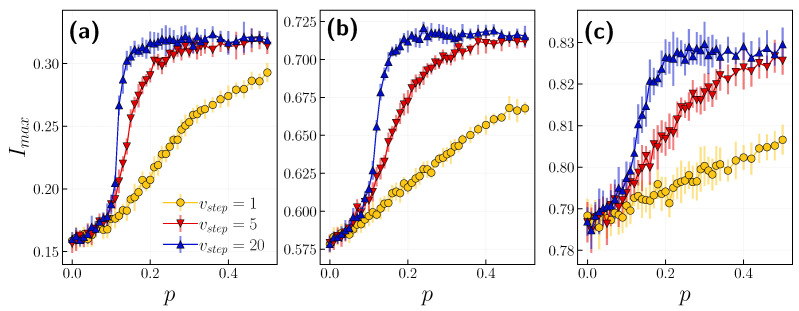
The peak of infection $I_{max}$ in function of independence probability *p* for selected timescales $v_{step}=\left\{1,5,10,20\right\}$. Each panel corresponds to a different infection probability, (**a**) β=0.02, (**b**) β=0.1, (**c**) β=0.5.

We now concentrate on the role of timescales with varying probabilities of independence. Figure 10 displays the relationship between the peak of infection $I_{max}$ and the independence probability *p* for different timescales $v_{step}$. We carry out simulations for three different β values. After surpassing p=0.2 for all infection probabilities, the infection peak only saturates if the speed of opinion update is greater than 10. Since the independence probability controls the noise in the system, the greater the *p*, the higher the peak of infection, and with faster opinion updates, we notice this effect earlier (see Figure 3).

**Figure 11:**
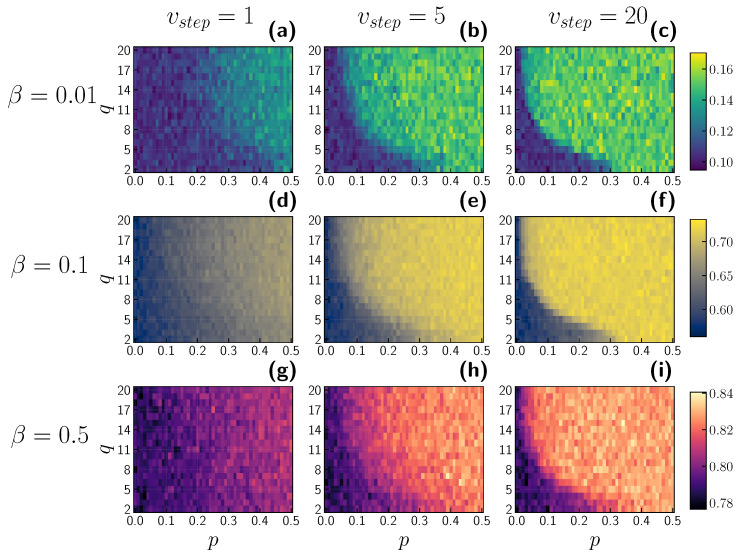
The peak of infection $I_{max}$ in function of independence probability *p* and group size *q*. Infection probabilities are shown on the left side of the panels. On top, the labels for $v_{step}$ are displayed. First, second and third row show the heatmaps for β=0.02,0.1,0.5 respectively. Panels (**a**,**d**,**g**) represent models with $v_{step}=1$, panels (**b**,**e**,**h**) with $v_{step}=5$ and panels (**c**,**f**,**i**) with $v_{step}=20$. Each pixel represents the average of 10 model realizations.

The whole picture of the interplay between the opinion and epidemic layer and the role of timescales is shown in Figure 11. We present heatmaps of the peak of infection with varying group sizes *q* and independence probabilities *p*. The first column comprises the results for $v_{step}=1$, i.e., both opinion and epidemic dynamics have the same timescale. One can observe that, for a lower independence probability, the peak of infection decreases, as seen in the results shown in Figure 4. With $v_{step}=1$, the dependence of group size *q* is not very noticeable. However, for $v_{step}=5$, i.e., when for one epidemic step, five updates on the opinion layer are performed, interesting patterns begin to emerge. Namely, when *q* is very large, we need to keep the independence probability low to reduce the peak of infection. In contrast, we are free to introduce a higher level of agents’ independence for smaller group sizes while maintaining an identical value for the infection peak. This phenomenon is more pronounced with greater $v_{step}$ (third column, $v_{step}=20$) and is not dependent on the infection probability β. In each of these two phases, the maximum infection value remains relatively stable. All these results are debated considering the population of agents with initial positive opinions, i.e., with $o_{init}=1$. On the other hand, in the case of a society with initially negative agents, one could assume the opposite conclusion. An increase in independence probability would introduce more significant noise in the system and, as a result, decrease the peak of infection. We presume a very similar behaviour as that in Figure 11, but with higher values in the bottom left regions and lower values elsewhere.

## 4. Discussion

In conclusion, we examine the interplay of opinion dynamics in the epidemic spread on a multiplex network, considering the role of timescales. Our model highlights the importance of people’s initial opinion towards restrictions, e.g., social distancing, wearing face masks, and hand washing. In the preliminary, early phase of the epidemic, the governing bodies should incorporate encouraging actions such as TV spots or advertisements to hinder the pace of the epidemic. The model shows that, with a positive public outlook, we can reduce the peak infection, even with many agents operating independently of the group, i.e., with greater *p*.

Agents who act independently and neglect all regulations could influence others to change their minds. As a result, society becomes fragmented, leading to a higher infection peak. It is worth mentioning that the size of the influencing group may restrict the level of individualism in the system. The group size could be larger for more conformist agents than for individualistic agents when maintaining the exact peak of infection. The interrelation between opinion and epidemic layers is significantly diminished with a higher infection probability. The difference between an individualistic society and a conformist society is less pronounced, i.e., the difference in the peak of infection does not exceed 0.1, whereas, for less infectious diseases, following a group could lead to a peak reduction of 0.2–0.4.

Another key aspect is the choice of proper timescales when simulating two dynamical processes. In our study, we introduce a simple mechanism of the relative rate of dynamics $v_{step}$, which results in the level of opinion being “faster” than the epidemic level. We need to recall that, as in all other opinion dynamics models, and the *q*-voter one, both the dynamics and the steady-state strongly depend on the initial conditions, i.e., the initial fraction of agents with positive opinions. If almost the entire society possesses a positive opinion, they have accepted the restrictions and now follow the rules imposed by the government agencies. On the other hand, a significant value for the independence probability *p*, which plays the role of noise in the system, ruins the opinion homogeneity and makes the society completely undecided, i.e., half of the individuals have positive and the other half have opposing opinions. For large values of $v_{step}$, the peak of the epidemic is higher than for the dynamics characterized by $v_{step}=1$. In the case of an “anti-restriction” society, when the majority of individuals ignore the epidemic, the existing conformity mechanism leads to an increase in the number of agents that do not respect the rules. In this setting, *p* is a “good” (from the government perspective) parameter, responsible for increasing the number of positive agents and suppressing the epidemic peak. As expected, by making the opinion dynamics faster than the epidemic dynamics, we increase the role of the opinion level. Therefore, we observe that the influence of the epidemic dynamic is less pronounced for the agents’ initial opinions.

We could have missed the relationship between independence probability *p* and group size *q*. However, on disparate timescales, this relationship started to appear (Figure 11). The best timescale in our model is unknown. To choose the best timescale $v_{step}$, one would need to incorporate empirical datasets with information on both opinion and epidemic dynamics. To the best of our knowledge, there is a lack of such datasets. The reason for this could be (i) difficulty in assessing people’s intrinsic opinion, (ii) difficulty tracking both the state and opinion of the person, (iii) difficulty obtaining knowledge of the entire contact network and also an online/virtual one. We want to underline that the selected dynamics of opinion and epidemic serve as examples of real phenomena. Undoubtedly, one could leverage different implementations of these dynamics, such as SIR, SIS or the majority voting model. We emphasize that, regardless of the choice of these two processes, we still need to carry out experiments to scrutinize the issue of timescales.

Finally, let us underline the possible extensions of this work. One could argue that the pace of opinion dynamics decreases over time. For instance, the views were altered multiple times at the beginning of the COVID-19 pandemic due to many unknown variables. Now, however, the opinion in society is crystalized, and only a few undecided people are changing their opinions. That fact might lead us to introduce varying opinion timesteps $v_{step}\left(t\right)$. Furthermore, one could take advantage of the available empirical datasets in two ways. First, these could be used to construct the underlying topology of the multiplex network, for instance, using a Bluetooth contact network as the epidemic layer and Facebook contacts as the opinion layer. Second, these could be used to collect surveys about people’s attitudes towards restrictions and use these as the initial fraction of positive agents. Moreover, we plan to reformulate the meaning of the opinion as views on vaccination and incorporate the political orientation of the agents. By leveraging the election dataset, one could study the correlation between epidemic spread and political views.

## Figures and Tables

**Table 1:** Model parameters with default values. Symbol ${}^{♠}$ indicates that a parameter could be changed during experiments.

Parameter	Default Value	Description
*N*	10,000 ${}^{♠}$	number of nodes
*m*	10	number of links generated by newly added node in network construction
$E_{add}$	0.01 Nm	number of additional links in opinion layer
*p*	0.01 ${}^{♠}$	probability of an agent to act independently in opinion layer
*q*	6 ${}^{♠}$	size of q-lobby in opinion layer
$o_{init}$	1.0 ${}^{♠}$	initial fraction of agents with positive opinions
$I_{init}$	0.1	initial fraction of infected agents
$t_{i}$	$x_{i}∼N\left(10,5^{2}\right)$	duration of infected state for agent *i*
β	${}^{♠}$	infection probability
γ	0.5	probability of an agent to enter the quarantine
μ	0.9	probability of recovery
κ	0.1	probability of death
$v_{step}$	1 ${}^{♠}$	number of opinion layer updates per one epidemic layer update

## Data Availability

Not applicable.

## Supplementary Materials

The following are available at https://www.mdpi.com/article/10.3390/e24010105/s1, Figure S1: Example multiplex visualization with the agents’ states at the beginning of the simulation. N=10,000, Figure S2: Example multiplex visualization with the agents’ states at the end of the simulation. N=10,000, Figure S3: Example multiplex visualization with the agents’ states at the beginning of the simulation. N=1000, Figure S4: Example multiplex visualization with the agents’ states at the end of the simulation. N=1000.
