# Supplementary material for: Role of Time Scales in the Coupled Epidemic-Opinion Dynamics on Multiplex Networks
Source: Entropy (Basel). 2022 Jan 9;24(1):105. doi: 10.3390/e24010105 (PMC8774805; doi:10.3390/e24010105)
Supplement: Supplementary file 1 [file entropy-24-00105-s001.zip › entropy-1509328-supplementary.pdf]

Supplementary Materials for Role of time scales in the coupled epidemic-opinion dynamics on multiplex networks

Robert Jankowski[1,2,3*] and Anna Chmiel[1]

[1]Faculty of Physics, Warsaw University of Technology, Koszykowa 75, PL-00-662 Warsaw, Poland
[2]Departamento de Física de la Materia Condensada, Universitat de Barcelona, Martí i Franquès, 1, E-08028 Barcelona, Spain
[2]Universitat de Barcelona Institute of Complex Systems (UBICS), Universitat de Barcelona, Barcelona, Spain
[*]Corresponding author. Email: robert.jankowski@ub.edu (RJ)

1 Multiplex visualization

In sample simulations, we set the following parameters: $p = 0.1$, $q = 6$, $\beta = 0.01$, $\gamma = 0.5$, $\mu = 0.9$, $\kappa = 0.1$, $o_{init} = 1$, $v_{step} = 1$. In the bottom (opinion) layer, the red color indicates a positive opinion ($+1$) whereas blue the negative one (-1). In the top layer (epidemic), the susceptible state is represented as green color, infected as orange, quarantined as yellow, recovered as purple, and dead as black.

In Figures S1 and S2 we present an example network representation before and after simulation, respectively, for network size $N = 1000$. Whereas in Figures S3 and S4 we show similar networks before and after simulation but with size $N = 1000$.

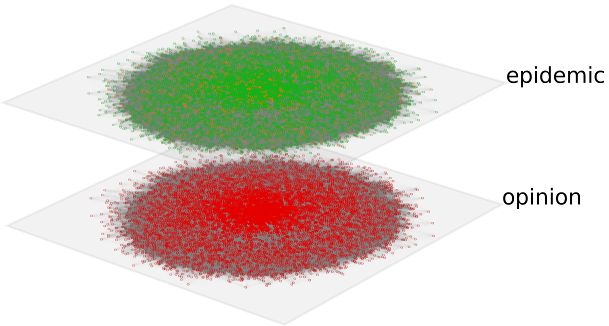

Figure S1: Example multiplex visualization with the agents' states at the beginning of the simulation. $N = 10000$.

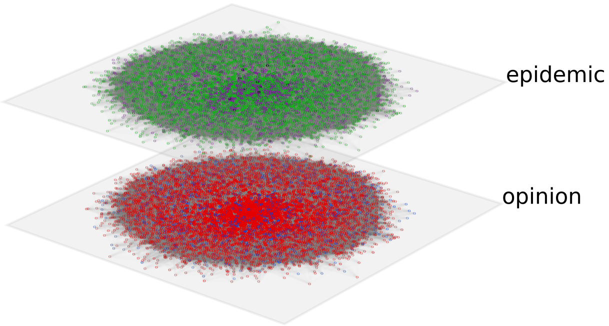

Figure S2: Example multiplex visualization with the agents' states at the end of the simulation. $N = 10000$

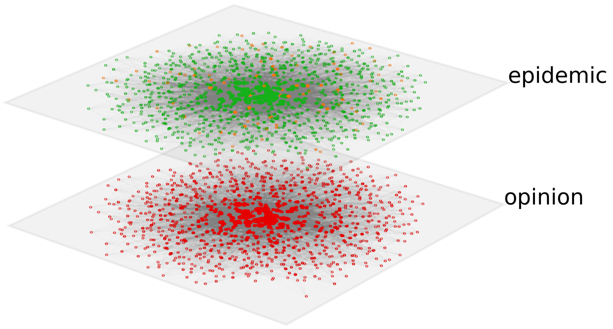

Figure S3: Example multiplex visualization with the agents' states at the beginning of the simulation. $N = 1000$.

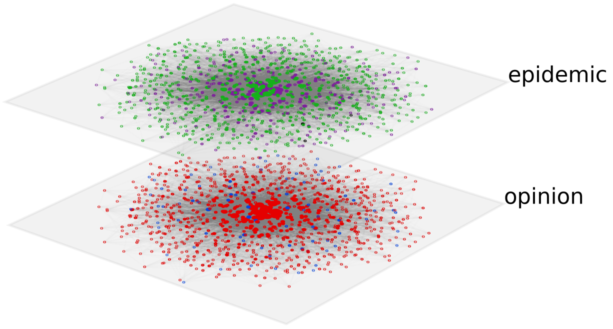

Figure S4: Example multiplex visualization with the agents' states at the end of the simulation. $N = 1000$.
